# The Timbre Perception Test (TPT): A new interactive musical assessment tool to measure timbre perception ability

**DOI:** 10.3758/s13414-020-02058-3

**Published:** 2020-06-11

**Authors:** Harin Lee, Daniel Müllensiefen

**Affiliations:** Department of Psychology, Goldsmiths, University of London, London, UK

**Keywords:** Timbre perception, Musical abilities, Musical assessment, Psychoacoustics, Gold-MSI

## Abstract

To date, tests that measure individual differences in the ability to perceive musical timbre are scarce in the published literature. The lack of such tool limits research on how timbre, a primary attribute of sound, is perceived and processed among individuals. The current paper describes the development of the Timbre Perception Test (TPT), in which participants use a slider to reproduce heard auditory stimuli that vary along three important dimensions of timbre: envelope, spectral flux, and spectral centroid. With a sample of 95 participants, the TPT was calibrated and validated against measures of related abilities and examined for its reliability. The results indicate that a short-version (8 minutes) of the TPT has good explanatory support from a factor analysis model, acceptable internal reliability (*α* = .69, ωt = .70), good test–retest reliability (*r* = .79) and substantial correlations with self-reported general musical sophistication (*ρ* = .63) and pitch discrimination (*ρ* = .56), as well as somewhat lower correlations with duration discrimination (*ρ* = .27), and musical instrument discrimination abilities (*ρ* = .33). Overall, the TPT represents a robust tool to measure an individual’s timbre perception ability. Furthermore, the use of sliders to perform a reproductive task has shown to be an effective approach in threshold testing. The current version of the TPT is openly available for research purposes.

## Background

Timbre is a primary perceptual attribute of complex sound, alongside pitch and loudness. Though, unlike pitch and loudness that are mainly related to a single physical parameter (i.e., frequency and sound intensity), timbre is a multidimensional attribute that arises from complex acoustic properties. It is broadly defined as *colour* or *texture* of an instrument (Helmholtz, 1954). Our ability to perceive such qualities from sounds enable us to discriminate a musical piece played by a buzzy trumpet from the same piece played by a mellow flute, even when both instruments are equal in loudness, tempo, and pitch (American National Standards Institute, 1994). Accordingly, timbre plays a key role in the recognition of sound sources because it is indicative of the event and action that triggered a sound (McAdams, 2013). Notwithstanding its importance, timbre remains a relatively poorly understood auditory attribute, presumably due to its multidimensional and complicated nature being a challenge in psychological timbre research.

Commencing with early works by Plomp (1970), Wessel (1973), and Grey (1977), who applied multidimensional scaling (MDS) of timbre (dis)similarity ratings, a lot of effort has been devoted to the identification of the dimensionality of the perceptual timbre space. Meanwhile several studies yielded a different number of potential acoustic correlates of timbre space dimensions, these days most researchers tend to agree that *attack time* and *spectral centroid* are the most salient timbral properties (Lakatos, 2000; McAdams, Winsberg, Donnadieu, De Soete, & Krimphoff, 1995; Siedenburg, Saitis, & McAdams, 2019). In addition, most researchers also agree that the development of the spectral composition of a sound over time constitutes an important dimension of timbre. *Spectral flux* or *spectral deviation* have been suggested as features to capture the developmental aspect of sound (McAdams, 2013), however, they still remain somewhat controversial attributes (see Caclin, McAdams, Smith, & Winsberg, 2005).

Attack time is defined as the duration a sound takes to reach its peak volume. For instance, bowing the string of a violin will produce a long attack time, whereas plucking the strings (*pizzicato*) will produce a short attack. The spectral centroid is defined as the relative weight concentration and the central tendency of a sound on the frequency spectrum (McAdams, 2019). Perceptually, the spectral centroid contributes to an impression of ‘brightness’ of a sound, generally ruling that a sound is perceptually brighter if the spectral centroid is positioned higher on the frequency spectrum (Schubert & Wolfe, 2006). The definitions of the other two potential features, spectral flux and spectral deviation, are more controversial. McAdams (2013) broadly defined spectral flux as the ‘degree of evolution of the spectral shape over a tone’s duration’ and spectral deviation as the ‘degree of jaggedness of the spectral shape’ (p. 41).

Some attempts have been made to analyze and measure spectral flux and spectral deviation using dedicated toolboxes (MIR toolbox by Lartillot, Toiviainen, & Eerola, 2008; Timbre toolbox by Peeters, Giordano, Susini, Misdariis, & McAdams, 2011), yet there is no single agreed descriptor underlying acoustic properties of these attributes. Perceptually, one way a variation of spectral flux can be distinctly perceived is by manipulating the phase alignment of the harmonic partials to induce spectral fluctuation (Zwicker & Fastl, 1999). When two tones with small frequency difference (<20 Hz) are presented simultaneously, known as beating frequency (Oster, 1973), the periodical alteration of constructive and destructive interference of sound waves gives rise to the phenonmenon of amplitude fluctuation (De Baene, Vandierendonck, Leman, Widmann, & Tervaniemi, 2004; Vassilakis & Kendall, 2010). This resulting amplitude fluctuation was described by Helmholtz (1954) as ‘roughness’, and it is perceptually characterized as impure or unpleasant sound qualities related to musical consonance (e.g., Plomp & Levelt, 1965). Therefore, in developing our test, we use roughness of complex sound as one of the testing timbre dimensions by implementing systematic deviation of the partials from the harmonic series to introduce amplitude fluctuation.

Despite the relative agreement on the importance of these discrete dimensions of timbre, only a few published auditory test batteries include a measure of timbral perception abilities. One such measure is the Timbre subtest from the Profile of Music Perception Skills (PROMS) test battery (Law & Zentner, 2012). In this test, the stimuli are designed using virtual orchestral library samples, and the trials progressively become more difficult, with the difficulty manipulated by the combination of instruments from the same, similar, or distantly related instrumental families. The participant’s task is to compare the two sounds and identify whether they are same or different instrumental combinations. Although this testing paradigm provides an ecologically valid approach by employing real orchestral instrument sounds, the acoustical properties of these instrumental combinations are not measured in any quantitative way. Consequently, it cannot provide practical information about an individual’s perceptual threshold as commonly provided in psychoacoustic tests. Furthermore, the test may be systematically biased towards classically trained musicians. For instance, the most challenging task of the test asks to compare a chord played by four violas with the same chord played by three violas and a violin. Musicians who have an extensive background with string instruments or have experience as instrumentalists in string quartets or orchestras are likely able to identify the subtle differences effortlessly, hence have an advantage on performing well on the test.

In today’s commercial music, nonacoustical instruments are widely used with multiple layers of complex sound-processing technology involved. Audiophiles, disk-jockeys, and sound engineers undergo years of training so that they can detect the finest details within synthetic sounds or mixtures of artificial and natural instruments. Yet these modern timbre perception experts may not have an ear attuned for combinations of string instruments; still, they may have an extraordinary ability to hear fine attributes of sound that most nontrained individuals might miss. In this respect, exclusively using orchestral instruments is a serious limitation to assess a wider audience when we consider how preferences and familiarity regarding Western orchestral music can differ between individual listeners.

The other auditory battery that includes tests related to timbre perception is the PSYCHOACOUSTICS toolbox (Soranzo & Grassi, 2014), which is a comprehensive MATLAB battery for testing auditory thresholds. It contains several tests for assessing thresholds, though they are not designed for the purpose of assessing timbre perception in any direct or comprehensive way. For example, the Duration Discrimination test in this toolbox can be considered as having a relationship with the amplitude envelope dimensions of timbre (see overview on ADSR envelope; Vail, 2014, p. 152). However, it measures individuals’ threshold in discriminating the *length of the notes* (which is only the sustain component of the envelope) rather than the *rise or fall duration of notes* (attack and decay components of the envelope) that are understood to be more salient timbral attributes (McAdams, 2019)*.* One other test from the toolbox that is worth mentioning is the profile analysis task, with which the idea was first introduced by Green (1983). Through series of experiments, Green and his colleagues (Green & Kidd, 1983; Green & Mason, 1985; Kidd, Mason, & Green, 1986) demonstrated that listeners can easily detect a small change in the intensity of a single component (i.e., a sinusoid) relative to the other components with equal amplitude (background). They argued that the listeners are able to detect the changes in the profile of the sound spectrum and perceive it as variations in ‘sound quality’. In this respect, although profile analysis may not directly fit into any of the described categories of timbre space dimensions, evidently it has strong relations with timbre perception.

The discussed auditory tests are useful in their own ways. However, to the best of our knowledge there are currently no existing tools that provide an empirical measure of individual differences in the ability to perceive and discriminate sounds along the perceptual dimensions of timbre. This largely limits our understanding of the underlying perceptual characteristics of sound and restrains progress in timbre research. Comparably, among the related fields of music perception, many tools have been developed over the past few decades to measure general and individual perceptual ability in pitch, loudness, and rhythm (e.g., Kidd, Watson, & Gygi, 2007; Peretz, Champod, & Hyde, 2003). Utilizing these tools, research on fine-grained pitch discrimination alone opened up new doors and shed a light on auditory cognition and interindividual musical abilities, involving research in absolute pitch (see review by Deutsch, 2013), congenital amusia (see review by Stewart, 2011), and children with autism (e.g., Heaton, Hermelin, & Pring, 1998), to only name a few. Therefore, in a similar repsect, developing a tool to measure individuals’ threshold in timbre perception empirically will greatly enhance future timbre research and enable the investigation of series of novel research questions. Ultimately, we can begin to disentangle the perception of what has been one of the most intricate aspects of sound.

We present a novel psychoacoustic assessment tool, the Timbre Perception Test (TPT), to fill the gap in the literature and to provide a robust measure that is specific to timbre and its three dimensions. This tool aims to examine perceptual abilities on three important dimensions of timbre (envelope, spectral centroid, and spectral flux) initially proposed by McAdams et al. (1995). By using synthetic sounds made of combination of sine waves, we avoid the potential bias of classical music training and the simultaneous influence of multiple timbral features that may covary when played in different registers and dynamics on acoustical instruments (Handel & Erickson, 2001). Furthermore, unlike existing tests that use alternative-forced-choice tasks or (dis)similarity ratings as response formats, we employ a production adjustment task using a new interactive software interface. We propose that this novel approach for testing avoids the dangers of attentional laps, affords shorter testing durations, and is highly engaging for participants.

The TPT was designed to measure participants’ ability to reproduce a heard sound as closely as possible by utilizing a movable slider as a method of average error that affects one sound dimension at a time. All participants were tested in two different conditions, with (a) unlimited playback opportunities (match trials) and (b) only a single playback (memory trials). In this study, we aim to determine whether both match and memory variants are largely relying on the same or different cognitive resources. Additionally, we investigate whether reproduction accuracy is reduced when playback is limited, and whether this is robust across a sample of participants differing in their musical training background. Golubock and Janata (2013) showed that working memory for unfamiliar timbre is relatively low, accordingly, we predict a considerably reduced accuracy when restricting the number of playbacks.

Although there is no direct evidence to suggest one’s ability for reproducing the qualities of timbre reflect their timbre perception ability, our view is that timbre perception is the crucial process for completing the TPT tasks. The importance of timbre perception ability for performing the TPT task becomes clear from the cognitive process model that we assume to underlie task performance: To complete a trial on the TPT, participants must first perceive the timbre of the target stimulus and subsequently hold a mental representation of this timbre in echoic memory (match condition) or in a working memory (memory condition). Subsequently, this is followed by iterative choices for the slider position to approximate the mental representation of the target with regards to the sounds produced via the test interface. On each of these iterations, participants need to make a judgement of perceptual closeness comparing the mental representation of the target timbre and the latest timbre just perceived and produced through the interface. As part of the iterative process, participants will acquire an understanding of the interface’s scale orientation and slider distances. Finally, once participants are not able to perceive any more differences between the target timbre and the timbre corresponding to the current slider position, they will decide to leave the slider at the current position and move to the next trial. Hence, timbre perception is assumed to be a core ability at all stages of the process model underlying the adjustment production task. Besides, the close relationship between perception task and production task have been shown for several other psychoacoustic and music domain: rhythm (Jacoby & McDermott, 2017; Sadakata, Desain, & Honing, 2006) and pitch (Liu, Jiang, Francart, Chan, & Wong, 2017).

A subsequent objective of this study is to assess the robustness of the new psychoacoustic test. Reliability is assessed by computing coefficients of internal consistency and test–retest correlation of test scores. Convergent validity is assessed by computing correlations between TPT scores and scores from related tests and self-report scales. We expect a positive correlation with performance on the Timbre subtest from the PROMS test battery, as well as positive correlations between the scores of the three TPT subtasks (Envelope, Spectral Centroid, Spectral Flux). We also expect to observe positive correlations between the three individual timbral dimensions of the TPT with related tests in the PSYCHOACOUSTICS toolbox that target (a) discrimination ability along the temporal dimension (i.e., Duration Discrimination test), (b) the centre of frequency dimension (i.e., Profile Analysis test), and (c) the pitch-harmony dimension (i.e., Pitch Discrimination test). However, correlations for these specific relationships are expected to be of smaller magnitude, given that the physical parameters and the perceptual dimensions targeted by the TPT and PSYCHOACOUSTICS toolbox measures are related but not identical. Finally, we expect to observe a strong relationship with the Goldsmiths Musical Sophistication Index (Gold-MSI) self-report inventory (Müllensiefen, Gingras, Musil, & Stewart, 2014), in particular with its subscales Musical Training, Perceptual Abilities, and the composite General Musical Sophistication scale. These would indicate that the TPT is indeed a measure of skilled musical expertise.

## Method

Ethical approval for the study was obtained from the Ethics Committee at the Psychology Department, Goldsmiths, University of London. Informed consent was obtained from all participants tested.

### Participants

Power analysis was conducted a priori to determine the number of participants required. Given our testing tool is a novel instrument and our primary interest is the correlations with a questionnaire and related tests, we decided to set .30 as the minimum effect size for observation. G*Power (Faul, Erdfelder, Buchner, & Lang, 2009) calculated that 84 participants would be required to achieve 80% power in a two-tailed, *p* = .05 correlational design.

A total of 104 participants (69 females) with a mean age of 25.21 years (*SD* = 9.26) were gathered from among the student population of Goldsmiths, University of London. To achieve a heterogeneous sample of participants with diverse musical backgrounds, the study was advertised to students in the music department and the psychology department. The overall sample mean of the Musical Training subscale was 26.96 (*SD* = 12.34) on the scale bounded at 7 and 49, which was comparable to the mean (*M* = 26.52, *SD* = 11.44) reported by Müllensiefen et al. (2014) from a large UK sample. Specifically, 26.5% reported to have more than 10 years of formal musical training, an equal amount reported to have no experience (26.5%). Subsequently the percentages were: 3 to 5 years (14.5%), 6 to 9 years (11.1%), 1 year (9.4%), and 2 years (6.8%).

Seven participants did not move the slider on more than half of the items of the TPT, and two participants’ data were missing for all items. Together, these nine participants were excluded from the analysis, and 95 sets of data remained for the final analysis. Participants were compensated for their time by either receiving course credits or a small monetary award.

### Development of the Timbre Perception Test (TPT)

The TPT aims to assess individuals’ perceptual ability to distinguish fine-grained timbral qualities in sound by assessing three important dimensions of timbre—namely, the amplitude envelope, spectral flux, and spectral centroid. The TPT was programmed using the MaxMSP software environment (Version 7.3.4, 64-bit, Cycling 74, San Francisco, CA) as a standalone application, which is portable for both Microsoft Windows and Mac OS operating systems (download available at www.osf.io/9c8qz). In the testing environment, these three dimensions were respectively labelled as Blocks 1, 2, and 3.

Eight sine-oscillators were used to produce sets of complex tone stimuli with one fundamental frequency (*f*_0_) and seven overtones. The overtones were multiples of whole number integers to the *f*_0_, starting from multiples of two to eight (i.e., first harmonic = *f*_0 ×_ 2, second harmonic = *f*_0 ×_ 3, etc.). The *stimulus tones* were repeated three times, indicated by a flickering blue light, at intervals of 800 ms. This repetition of the tones was to ensure that participants hear the stimuli during memory trials, in which the playback is limited.

Five pitch-tones were employed with notes ranging across two octaves (from G3 to A#4) to encompass a wide range of frequency spectrum. Moreover, five acoustic values of attack/decay, spectral flux, and spectral centroid were mix-matched to produce five unique parameter sets. These sets were mapped on to the stimuli and systematically organized to ensure that all five sets are presented for every testing dimension (in a varied order). The full acoustic range of each testing dimension and parameter values used for the stimuli are reported in Appendix C.

Unlike the stimulus tones, the participants could manipulate the *reproduction tone* by moving an interactive slider (with a slider range of 0–100) to change the sound profile according to the dimension being tested, whereas the other two dimensions not being tested had identical profiles to the stimulus. For instance, when participants performed a trial in manipulating attack/decay (here group termed as ‘Envelope’), moving the slider only affected the envelope of the reproduction tone, whereas the parameters of spectral flux and spectral centroid remained unchanged (i.e., identical profiles to the corresponding stimulus tone).

**Fig. 1:**
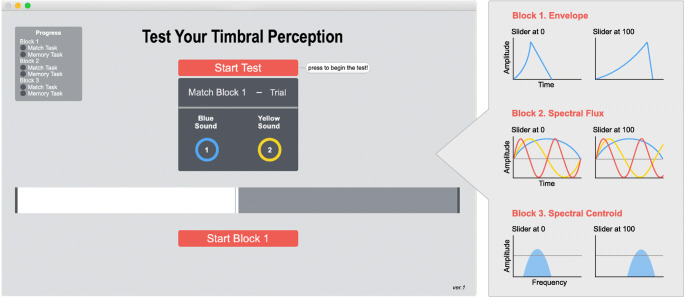
The layout of the TPT (left) and its testing dimensions (right). Graphic figures for the testing dimensions show how the reproduction tone is manipulated when the slider is positioned at ‘0’ (far left) or positioned at ‘100’ (far right). Envelope represents rise and fall time in amplitude, Spectral Flux represents the alignment of harmonics that results as more consonant when aligned in-phase, Spectral Centroid represents the filtered frequency area in the frequency spectrum. (Colour figure online)

Ultimately, the participants’ task was to manipulate the reproduction tone by moving the slider to replicate the stimulus tone as accurately as possible. Figure 1 illustrates the layout of the TPT software and graphical representation of the change in sound profiles of the subtasks by the movement of the slider.

#### Testing parameters

In the Envelope subtask (Block 1), the slider bar altered the log attack time which also inversely influenced the decay time of the reproduction tone. Log attack time has shown to be the salient attribute of timbre identification, whereas lesser extent for the decay time. Nonetheless, we included the decay time to keep the total duration of the stimulus approximately constant and allow listeners to focus on the interplay between the two parameters. We reasoned that if only the attack time was included, there would be a potential risk of participants judging the stimulus merely by the total tone duration instead of by its dynamics of the rise and fall in amplitude. Hence, moving slider to the left (i.e., closer to zero) manipulated the reproduction tone to have a shorter attack with longer decay time, whereas moving the slider to the right (i.e., closer to 100) resulted in longer attack with shorter decay times, with them always having an inversely proportional relationship. The full acoustic range covered by the slider in each subtask is reported in Table 1.

**Table 1:** Parameters of the three subtasks of TPT with theoretical slider range from 0 to 100

	Envelope (ms)		Spectral Flux (multiples to the *f*_0_)				Spectral Centroid (Hz)
	Attack	Decay	4th harmonic	5th harmonic	6th harmonic	7th harmonic	
Slider range (0–100)	5–291	50–5	3.0–3.3	4.0–3.7	5.0–5.2	6.0–5.8	600–1k
Link function	Log base of 1.03		Linear				X^2^

*Note*. Attack and decay, 4th & 5th and 6th & 7th pair of harmonics have inversely proportional relationships. X = slider value/100. ‘Link function’ describes the relationship between the physical parameters of the sounds and slider scale of 0–100

In the Spectral Flux (Block 2) subtask, the ratios of harmonics to the fundamental frequency were altered to introduce dissonance caused by the beatings of frequency, characterized as ‘roughness’. To achieve this effect, four harmonics were manipulated with the movement of the slider. This manipulation occurred by altering the ratio between the harmonics and their whole-number integers (i.e., when the slider was moved from left to right, the ratios of the 4th and 6th harmonics were increased and those of the 5th and 7th harmonics were decreased). Similar to the Envelope subtask, the inversely proportional relationship between two pairs of harmonics was to prevent participants from making judgments based merely on the rise or fall in global pitch. Moving slider to the left aligned the harmonics closer to the whole integer numbers and therefore more consonant. Meanwhile, moving to the right introduced more dissonance as the number of beating frequencies increased.

In the Spectral Centroid subtask (Block 3), a bandpass filter was applied to the source sound to alter its spectral centroid, which has shown to be a good predictor of the perceptual ‘brightness’ of a sound. The bandpass filter is characterized by two main components: one being the ‘centre frequency’ (also known as ‘resonant frequency’) which is the peak frequency response, and the other being the quality factor ‘Q’ which describes the ratio of the centre frequency to the bandwidth. Higher Q value corresponds to the passing of narrower frequency spectrum, resulting as a pointier bell-shaped curve when observed with an audio equalizer spectrum. For this subtask, Q remained constant at a ratio of 1.8 and only the centre frequency was manipulated. Positioning the slider from left to right moved the centre frequency of the sound from low to high on the frequency spectrum, with brighter sounds located on the right. The filter responded to the slider following a logarithmic relationship in agreement with the basic principle of human frequency perception (Moore & Glasberg, 2007).

#### Pilot testing

To establish suitable parameter ranges for the subtasks, pilot testing (*N* = 15, 10 females; age *M* = 27 years, *SD* = 6.8) was conducted to assess the level of difficulty of the items. In the first instance, we tried testing a few participants on a version of the task that combined all three dimensions of timbre (i.e., simultaneous manipulation of three sliders). However, almost all participants found it very difficult to get a good understanding of the task, and we could not judge whether they were attending to the changes produced by each slider. Hence, we decided subsequently to simplify the interface by splitting the full experiment into three subtasks, with each subtask only presenting one slider (i.e., manipulating only a single timbre dimension at a time). The pilot test consisted of four trials per subtask without restricting the playback of the stimuli. Judging by the absolute distance of participants’ slider position from the target value, the results indicated that the Envelope and Spectral Centroid subtasks were relatively easy compared with the Spectral Flux subtask. Therefore, the parameters were adjusted to balance the level of difficulty across the subtasks.

Subsequently, a second pilot test was conducted by reinviting six of the participants from the first pilot test. The distribution of responses confirmed that the difficulty of the three tasks roughly matched in terms of the absolute distance to the target value of the stimulus, with Envelope (*Mean absolute slider distance from target* = 15.0 points, *SD* = 11.9), Spectral Flux (*M* = 20.1 points, *SD* = 17.7), and Spectral Centroid (*M* = 15.7 points, *SD* = 15.7). These new parameter ranges as given in Table 1 were used for the main experiment.

#### Final calibration

Participants took approximately 5 minutes to complete the full pilot test. Given such short duration, an extra item was added on each subtask, as well as ten trials with limited playback (*memory trials*). The memory trials differed from the here-called *match trials* in that the stimulus sound could be played-back only once at the beginning of a trial. The participants had to retrieve the heard attributes of the timbre and adjust the slider entirely from memory. Thus, the final version of TPT for the main experiment comprised of five items of match trials and ten items of memory trials for each of the three subtasks (Envelope, Spectral Flux, and Spectral Centroid) presented in blocks, totalling 45 items. In addition, a training item was included prior to beginning each subtask for participants to become familiarized with the changes that it produced. The final version for the experiment lasted about 10–15 minutes.

### Materials for testing validity

#### *Pitch discrimination of complex tones (Soranzo & Grassi,*^2014^)

This test is part of the PSYCHOACOUSTICS toolbox for MATLAB and is designed to examine listener’s threshold in detecting differences in two pitches. It employs a three-alternative forced-choice (3AFC) response paradigm in which three complex tones are presented to the listener in quick succession. Two of the complex tones are played back with the base frequency 330 Hz, while one is higher in pitch (starting frequency at 390.01 Hz). Participants have to identify which one of the three sounds is highest by indicating with number 1, 2, or 3 on the keyboard. In our experiment, participants performed the task using the maximum likelihood procedure (MLP; Shen & Richards, 2012) with two blocks and 30 trials per procedure (blocks averaged for analysis), taking about 4 minutes in duration. The MLP method have been employed extensively in auditory threshold testing for clinical trials (e.g., Benoit et al., 2014; Flaugnacco et al., 2014) and validating newly developed listening tests (e.g., Larrouy-Maestri, Harrison, & Müllensiefen, 2019).

#### Duration discrimination of complex tones (Soranzo & Grassi, 2014)

The test is part of the PSYCHOACOUSTICS toolbox and measures the listener’s perceptual threshold in detecting duration of musical notes. Three complex tones are presented to the listener with two having note lengths of 250 ms while one having a longer length (starting length at 450 ms). Listeners have to identify the longest tone and it followed the same testing procedure as the pitch discrimination test, taking about 4 minutes in duration.

#### Profile analysis (Soranzo & Grassi, 2014)

The test is part of PSYCHOACOUSTICS toolbox and measures the listener’s perceptual threshold in detecting amplitude variation of harmonics in a complex tone. Three complex tones are presented to the listener with two having 5 harmonics with fixed amplitude of −4.0 dB while one having a higher amplitude for the 3rd harmonic (starting amplitude at 20 dB). Listeners have to tell the odd sounding tone. Due to the MLP option being faulty for the particular task, the test was run with the Staircase stimulus selection method for a single block with 3AFC, two-down-one-up, 8 reversals, taking about 6 minutes in duration.

#### Timbre subtest from the Profile of Music Perception Skills battery (PROMS; Law & Zentner, 2012)

In this test, stimuli are generated using a virtual sound sample library, consisting of chords of four notes (C4, E4, G4, C5) lasting 1.5 s in length, taking about 8 minutes to complete a total of 18 trials. Participants compare whether the stimuli are played by identical instruments or not by responding on a scale from 1 (*definitely different*) to 5 (*definitely same*). For the easy trials at the beginning of the test, when comparing nonidentical instruments, the instruments are from different families (e.g., horn vs. strings). However, trial by trial, the test gradually becomes more difficult as the comparison is made between similar or within the same instrument family (e.g., most difficult trial compares four violas with three violas and a violin). Individuals’ score is calculated by assigning a score of 1 for a corrected response, 0.5 for a partially correct (i.e., probably different or probably the same), and 0 for an incorrect response. These scores are summed together with the highest possible score being 18. The original study (*N* = 56) for the Timbre subtest reported a mean raw score of 11.92 (*SD* = 3.12), internal consistency of *α* = .77 and ω = .73, and test–retest reliability of *r* = .69 (with subsample of *n* = 20).

#### Gold-MSI self-report questionnaire (Müllensiefen et al., 2014)

This short questionnaire addresses several aspects of musical expertise and engagement, comprising 39 items on five subscales (Active Engagement, Emotions, Musical Training, Perceptual Abilities, and Singing Abilities) and a General Musical Sophistication score. From the original study, comparison data is available from a very large sample (*N* = 147,663) representing the general, nonspecialist population.

### Procedure

Testing took place in isolated cubicles with Windows 10 operating computers and the stimuli were presented using Behringer HPM-1000 headphones (Behringer GmbH, Willich, Germany). MATLAB (Version R2018a) was used to run the tests from the PSYCHOACOUSTICS toolbox (Soranzo & Grassi, 2014).

The test battery consisted of six assessments and progressed in the following order: hearing assessment, TPT, Pitch Discrimination, Duration Discrimination, Profile Analysis, Timbre subtest from PROMS, and Gold-MSI self-report. After signing the informed consent, a short online hearing assessment^1^ based on a speech-in-noise hearing test was conducted to screen out participants with impaired hearing. None of the participants in our sample fell below the clinical threshold of 70% correct-response rate. Subsequently, participants received verbal instructions on how to perform the TPT along with the interactive speech bubbles that appeared on the screen during the first training trial.

Participants completed each trial by first listening to the stimulus tone and then by moving the slider bar to adjust the reproduction tone to replicate the stimulus tone as closely as possible. For ease of playback, keyboard shortcuts were used to play the stimulus (keypad ‘1’) and reproduction (keypad ‘2’) tones. They were encouraged to compare the two sounds as many times as necessary during the match trials, whereas they were informed that the stimulus is played only once in the memory trials (if participants clicked the stimulus sound during a memory trial, a speech bubble appeared stating “Remember you can play back the blue sound only once during the memory task!”).

Participants were also informed at the beginning that they would proceed through three separate blocks of tasks with each block consisting of a test trial, five matching trials, and ten memory trials. The overall progress could be tracked with the progress bar, but they were not given any information with regards to how the sounds and the meaning of the slider changed for each block.

Subsequently, participants performed three tests from the PSYCHOACOUSTICS toolbox within the MATLAB environment and Timbre subtest from the PROMS test battery online. Lastly, they were asked to fill the Gold-MSI self-report questionnaire online and were thanked for their contribution. The full test battery lasted about 1 hour in duration.

## Results

Our primary analysis goal was to assess whether all three subtasks, targeting different dimensions of timbre and in their variants as matching and memory trials, are measuring the same or different cognitive abilities. A subsequent goal was the assessment of the TPT’s reliability, and its validity with related tests and questionnaires. Given this aim, the analysis process was carried out in the following stages: (1) Raw scores (i.e., absolute distances between participants’ slider positions and the target value) were binned for every item of the TPT to generate performance scores for individual participants. (2) These performance scores were averaged at the level of subtasks and analyzed by computing correlations across all subtasks and subsequently using factor analysis. (3) The final TPT scores and their match and memory variants were examined for reliability using Cronbach’s alpha, McDonald’s Omega, and test–retest correlations with 1–2 weeks of interval. (4) Using correlational analyses, validity of the TPT was evaluated against existing tests that measure related perceptual abilities and self-reported musical expertise. (5) Accuracy in reproducing ability was compared for conditions of unlimited playback and limited playback.

All analyses were performed using the R software, specifically the R packages ‘psych’ (Revelle, 2019), ‘dplyr’ (Wickham, François, Henry, Müller, & RStudio, 2019), and ‘Hmisc’ (Harrell, 2019). Descriptive statistics of the full test battery are reported in Appendix A. The data sets for all experiments are available online (https://osf.io/mkj8f/).

### Bin scoring

Participants’ raw scores for individual items were defined as the absolute value of the chosen slider position on the 0 to 100 scale minus the correct value of the target stimulus tone presented. Raw scores were converted into bin numbers, with bins having roughly equal numbers of observations and varying widths on the slider scale representing the physical attribute being manipulated (see Appendix C for bin ranges and corresponding acoustical properties). This binning procedure was used as a nonparametric technique to standardize the scores across the three testing dimensions, as well as to smooth the raw data that was assumed to contain measurement noise. One other important reason for the binning was to allow for approximate mapping of a physical scale on to a perceptual scale that is monotonically related, provided that we cannot assume our slider scale range (0–100) to map linearly onto the perceptual scale of listeners (e.g., listeners’ perceptual scale and the physical slider scale may have a logarithmic relationship or any other nonlinear but monotonic relationship).

Sliders kept at default position were not treated as missing values because participants could have intentionally left the slider untouched as they perceived the reproduction tone to be already close enough to the target. However, we set a criterion threshold to exclude any items that had more than 30% observations with the sliders left unmoved. This threshold ensured that a sufficient number of bins (with roughly equal numbers of observations) could be computed for each item, ensuring a good discriminatory power of each item. One item from the Envelope subtask with 35% of the observations at default position and one item from the Spectral Flux subtask (33% at default) were excluded from the analysis on the basis of the a priori threshold criterion.

Considering the total number of participants and the rates at which the slider was not moved across trials, we decided to use six bins for all items across all subtasks. Using six bins represented a good compromise between measurement resolution and a balanced number of observations across bins. Bins were assigned integer numbers and bin numbers were used as the basis for each participant’s bin score for individual items of the TPT, with 6 being the best and 1 being the worst performance bin. The scores were then aggregated by averaging across items for each of the three subtasks in their match and memory variants. In addition, the overall means for the memory and match variants were computed (see Appendix A).

### Factor analysis

**Fig. 2:**
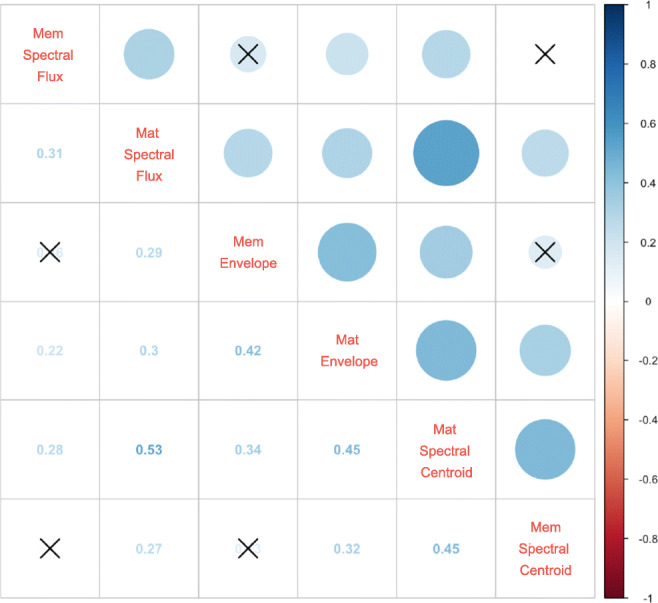
Spearman’s correlations between six score variables of the TPT. The size of blue circles represents the magnitude of the correlations, and crossed circles represent statistically nonsignificant pairs at a threshold of *p* = .05. Mat = matching variant of subtask; Mem = memory variant of subtask. (Colour figure online)

Exploratory factor analysis was conducted to assess whether all three subtasks of the TPT targeting different physical parameters of timbre can be summarized to measure the same construct, and whether the memory and match variants reveal the same or different factors. Initially, we assessed factorability of the three subtasks of the TPT separated into match and memory variants (totalling six score variables). First, it was observed from the Spearman’s correlation matrix of the TPT’s subtasks that all scores were correlated significantly by *ρ* > .30, with at least two other scores (see Fig. 2). Second, the Kaiser–Meyer–Olkin measure of sampling adequacy was 0.74, which is higher than the commonly accepted threshold value of 0.60. Bartlett’s test of sphericity was significant at χ^2^(15) = 123.07, *p* < .001. Given these overall indicators, the set of all subtask scores of the TPT was deemed suitable for factor analysis.

We ran an exploratory factor analysis, using the minimum residual method, given our interest was to examine whether there were one or more cognitive constructs underlying the TPT scores on the six subtasks, and whether these constructs can be summarized separately or uniformly by match and memory variants of timbre perception. Kaiser’s criterion of eigenvalues >1 and parallel analysis both suggested a single factor solution. Loadings on the single factor were highest for the matching variants of the subtasks (Spectral Centroid = 0.84, Spectral Flux = 0.64, and Envelope = 0.59), while considerably lower for the memory variants (Envelope = 0.50, Spectral Centroid = 0.41, and Spectral Flux = 0.33). This implied that the memory variants may be not suitable for measuring the timbre perception and reproduction ability, at least not to the same degree and within the same model that describes the performance on the matching variants of the subtasks.

Thereby we examined a two-factor solution, allowing for the match and memory variants of subtasks to load potentially on separate factors. However, even in the two-factor solution, all match and memory variants of the three subtasks had higher loadings on Factor 1 (loadings > .30) than on Factor 2, except for the memory variant of the Envelope subtask that loaded very strongly (loading = 0.99) on Factor 2. Furthermore, the loadings of memory variants on Factor 1 were again relatively weak in comparison to the loadings of the match variants. Hence, these patterns of factor loadings suggested again that the match trials of the TPT are coherently measuring the same cognitive ability, whereas the memory trials appear to form a more heterogenous set while also being less strongly associated. Furthermore, the two-factor solution produced worse fit indices, both in terms of absolute (*RMSEA* = .079) and relative fit (*BIC* = −13) than the single factor solution (*RMSEA* = .075, *BIC* = −27).

Given this pattern of results, a single factor solution was deemed more suitable to explain the common variance among the subtasks of the TPT. Due to the low loadings of the memory variants of all three subtasks, we computed another minimum residual factor analysis specifying a single factor and using only the match variants of the subtasks as input variables. This final factor solution explained 48% of the variance, which is the best absolute fit of the factor models we tested, and all three variables showed high loadings on the single factor: Spectral Centroid (0.91), Spectral Flux (0.60), and Envelope (0.50). Hence, the single factor model using only the 13 match items represents an internally coherent model. As explained in the Discussion section below, this brief version of the TPT is recommended for use in practical situations where time is limited and the aim is to assess individual differences in timbre perception, while ignoring timbre memory.

### Reliability

Reliability of the full TPT score and its match and memory variants were assessed by computing internal consistency/reliability and test–retest reliability. While Cronbach’s *α* is most commonly reported as coefficient for internal reliability, it assumes equal loadings of all item (i.e., tau-equivalence), and therefore likely to be violated in our data. Thus, we also report McDonald’s omega as the alternative index of internal consistency, which is based on the hierarchical factor model and more appropriate for our design. Evidently, as tau-equivalence was not met, values of the two kinds of reliability coefficients (computed across all items) diverged considerably for the full TPT test (*α* = .74, ωt = .80) and the subset of only memory items (*α* = .50, ωt = .76), but less so for the subset of match items (*α* = .69, ωt = .70).

Test–retest reliability was independently assessed among 25 new participants (a mean interval of 7.1 days, *SD* = 3.8). Following the results from factor analysis, we only assessed the test–retest reliability of the short version of the TPT that excludes the two match items with an unbalanced distribution of responses as well as all memory items, leaving a total of 13 match items. Raw absolute slider distances to the TPT scores conversion followed the pre-established bin boundaries from the main test (see Appendix C for bin ranges for individual items). A two-way random effect model with absolute agreement definition was used to measure intraclass correlation. The resulting test–retest reliability coefficients were in good to acceptable range according to common standards, *ICC* (24) = .79, *r* = .79, *ρ* = .75; all *p*s < .001.

### Validity

Normality of scores was assessed for tests of the full test battery by interpreting Q-Q plots as well as the Shapiro–Wilk normality test using the *p* > .05 criterion. All components of the TPT and Timbre subtest from the PROMS battery were normally distributed, whereas all except Active Engagement subscale of the Gold-MSI and all three tests from the PSYCHOACOUSTICS toolbox did not follow a normal distribution. Given that a considerable number of variables were not normally distributed and that scores of the TPT are ordinal, Spearman’s correlation coefficients was considered suitable. Moreover, since multiple comparisons were carried out, Benjamini and Hochberg’s (1995) *p*-value correction was applied to set a stricter criterion for accepting correlations as statistically significant. Table 2 shows the correlations between TPT and the other measures in the battery. In addition, correlations between the Timbre subtest of the PROMS, subscales of the Gold-MSI self-report inventory, and three tests from PSYCHOACOUSTICS can be found in Appendix B.

**Table 2:** Spearman’s correlations of the TPT with the convergent validity measures

	Gold-MSI						PSYCHOACOUSTICS^1^			PROMS (timbre)
	G0	G1	G2	G3	G4	G5	Pitch	Duration	Profile	
Match Envelope	.43***	.38***	.46***	.40***	.33**	.47***	.41***	.19	.00	.13
Memory Envelope	.49***	.28**	.45***	.31**	.26*	.44***	.38***	.19	.06	.18
Match Flux	.39***	.42***	.51***	.33**	.36***	.48***	.43***	.11	.16	.26*
Memory Flux	.23*	.36***	.39***	.14	.20	.34**	.28*	.08	.08	.13
Match Centroid	.30**	.42***	.36***	.34**	.26**	.37*	.40***	.27*	.07	.33**
Memory Centroid	.20	.26*	.27**	.11	.12	.27**	.22*	.28*	.07	.19
Match Total	.50***	.54***	.61***	.50***	.42***	.60***	.54***	.28*	.14	.36**
Memory Total	.50***	.47***	.59***	.30**	.30**	.56***	.49***	.22*	.12	.25*
Overall Score	.52***	.56***	.64***	.45***	.40***	.62***	.56***	.27*	.15	.33**

*Note.* G0 = Active Engagement; G1 = Perceptual Abilities; G2 = Musical Training; G3 = Singing Abilities; G4 = Emotions; G5 = General Sophistication

^1^Threshold of tests from PSYCHOACOUSTICS were calculated by taking the average of blocks converted into log values

*p < .05. **p < .01. ***p < .001. Significant levels are adjusted according to Benjamini and Hochberg (1995)

Additionally, correlations between the General Sophistication score of the Gold-MSI and the TPT by the number of trials were examined to determine how many trials are required to reach a plateau.

**Fig. 3:**
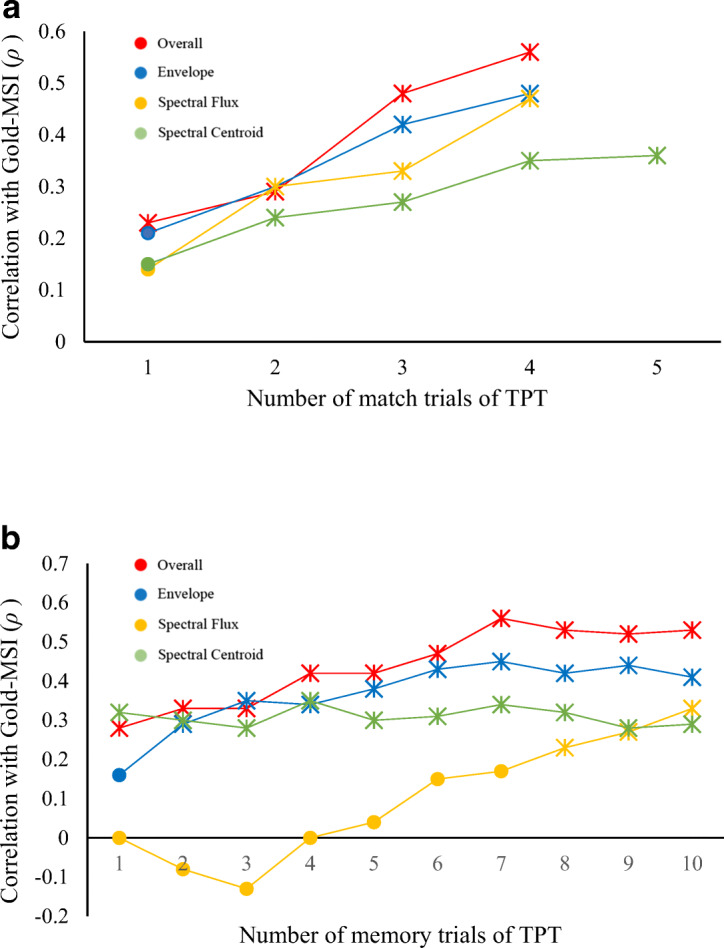
**a** Trial-by-trial correlations between number of TPT *match trials* and Gold-MSI General Sophistication (G5). **b** Trial-by-trial correlations between number of TPT *memory trials* and Gold-MSI General Sophistication (G5). *Note*. X symbol represents significance at *p* < .05. (Colour figure online)

Figures 3a show a steady increase in correlations over the increasing number of trials for the match variants (estimated to reach plateau by 5–6 trials at a correlation level of about *ρ* = .60). In contrast, Fig. 3b shows that a plateau—though at a substantially lower level—was reached earlier (2–3 trials, correlation level of *ρ* = .35) for the memory variants.

### Match versus memory

Raw absolute distance scores (on the 0–100 slider scale) and their corresponding acoustical parameter values were used to compare the match and memory conditions. Table 3 illustrates the mean reproduction accuracy by the absolute slider distance from the target and the corresponding acoustical parameter values. Considering the mean absolute distance from target, the results clearly indicate that participants found the Envelope subtask to be the easiest for both match and memory tasks, while the Spectral Flux and Spectral Centroid subtasks were of comparable difficulty. Moreover, accuracy in reproducing the heard sound was reduced across all subtasks when the stimuli was restricted to a single playback. Both the Envelope and Spectral Flux subtasks fell in accuracy by a slider distance of about 5 points, whereas a smaller reduction of 3.6 was observed for Spectral Centroid.

**Table 3:** Absolute distance and corresponding acoustical thresholds of testing dimensions of TPT

	Match condition			Memory condition		
	Envelope	Spectral Flux	Spectral Centroid	Envelope	Spectral Flux	Spectral Centroid
Mean abs slider distance	9.65 (10.23)	17.93 (16.98)	20.02 (15.64)	14.68 (13.09)	23.24 (17.54)	23.69 (17.87)
(*SD*)						
Mean acoustical threshold	14.41 ms (18.28 ms)	0.0500 β^1^ (0.0391 β)	68.47 Hz^2^ (63.89 Hz)	18.81 ms (22.31 ms)	0.0581 β (0.0439 β)	95.24 Hz (74.53 Hz)
(*SD*)						

^1^β = arithmetic mean deviation in ratio of four harmonics from their original whole number integer. Absolute distance is calculated by | target value–position of slider | with a theoretical slider range of 0–100

^2^Reference frequency is 700 Hz at slider position = 50

Finally, the total performance score of the TPT was related to the participant’s total number of playbacks of target stimulus tones (*ρ =* 0.43, *p* < .001) and reproduction tones (*ρ =* 0.36, *p* < .001) across all trials of the match subtasks. We also considered the possibility that participants who are more musically trained may have applied more effort in adjusting the two tones. However, we found only a very small and nonsignificant relationship between number of stimulus (*ρ* = 0.14, *p* = .21) and reproduction (*ρ* = 0.09, *p* = .41) tone playbacks with subscale Musical Training of the Gold-MSI.

## Discussion

We developed the TPT with the aim to provide a tool to the research community for measuring individual differences in timbre perception ability. Timbre is a primary auditory attribute commonly understood to have a multidimensional structure (Grey, 1977). In light of the existing literature on the dimensions of the timbre space (Caclin et al., 2005; McAdams, 2019), the TPT aims to measure an individual’s ability in reproducing three important dimensions of timbre–namely the amplitude envelope, spectral centroid, and spectral flux. Moreover, we assessed the role of memory within each dimension by comparing match (unlimited playback of stimuli) and memory (stimuli only heard once) variants of all three subtasks. For the implementation of the TPT, we employed a graphical user interface (GUI) featuring a slider as a method of average error, in which participants can manipulate their reproduction sound along a given dimension. This production paradigm distinguishes the TPT from traditional testing paradigms for timbre perception which predominantly rely on (dis)similarity ratings or same–different judgements.

With a sample of 95 participants, the TPT was validated against related tests and examined for its reliability. Two different factor models indicated that the match variants of all three subtasks loaded on a single factor, implying that they are measuring the same cognitive ability. However, memory variants showed heterogeneous and weaker factor loadings, suggesting that they should not be included in the same model with the match variants. Interpreting these results, we excluded all memory subtasks as well as several items from the match variants with low discriminatory power to construct a short version of the TPT. This short version of the TPT showed acceptable level of internal consistency according to the common standard (Cortina, 1993) and good test–retest reliability.

The validity of the TPT was assessed by computing correlations with the Timbre subtest of the PROMS test battery, three associated tests from the PSYCHOACOUSTICS toolbox, and Gold-MSI self-report inventory. The moderate but significant correlation between the TPT composite score and Timbre test from the PROMS battery supported the validity of the TPT. However, the correlations of the TPT with the PROMS timbre test was weaker than the correlations between TPT and the Gold-MSI self-report subscales assessing general musical expertise. The only moderate correlations between TPT and the PROMS timbre test may have been caused by the different nature of the tasks (reproduction on a continuous scale vs. binary discrimination). Indeed, it has been suggested that the interpretation of different threshold measures obtained by force-choice paradigms and manipulation tasks can be problematic (Turner, Horwitz, & Souza, 1994). Unfortunately, to our knowledge there exists no other individual differences test measuring timbre perception or timbre memory, therefore it is not feasible at this stage to assess which of the two tests is more valid measure of timbre perception and processing ability. In any case, from the current dataset, the TPT showed considerably larger correlations with the self-reported measure of Perceptual Ability from the Gold-MSI as well as with all perceptual tests from the PSYCHOACOUSTICS toolbox (see table in the Appendix B), which suggests that the newly developed test may be measuring aspects of timbre perception that the PROMS is not capturing.

On the other hand, the correlations with the tests from the PSYCHOACOUSTICS toolbox show a somewhat complex picture. Supporting the TPT’s validity, the Pitch Discrimination test from the PSYCHOACOUSTICS toolbox was substantially correlated with the associated performance on the Spectral Flux subtask as well as most other TPT subtasks, and particularly strongly with the TPT total scores. This suggests that the ability to discriminate pitch is associated with the perception of spectral flux, which was implemented through the manipulation of the harmonics of a complex sound. However, Duration Discrimination did not reveal statistically significant associations (after adjustment for multiple testing) with the expected TPT’s Envelope subtask. The lack of correlation between Duration Discrimination (comparison of the length of individual tones) with Envelope (rise and decay time of tones) may imply that recognizing the temporal dynamics of a sound is a different cognitive ability to recognizing the duration of tones, which may only require a simpler temporal judgment. To our surprise, the Profile Analysis test did not correlate with the expected Spectral Centroid subtask of the TPT nor with any of the tests within the battery. The discrepancies may have risen from Profile Analysis being rather a qualitative task in nature as argued by Green and Kidd (1983), whereas the TPT involves a quantitative measure of perceptual thresholds. Still, this cannot explain why the Profile Analysis showed no relationship with the PROMS (both being qualitative) and further investigations is required.

The TPT and its individual subtask components revealed strong correlations with all subscales and the composite score of the Gold-MSI. As we hypothesized, among these subscales, ‘Musical Training’ and ‘Perceptual Abilities’ showed the strongest correlations. The results make intuitive sense as self-reported ability in musical perception, if accurate, should correspond to the performance on tests of listening ability. Besides, the amount of musical training has consistently been shown to be the main factor influencing the performance on musical ability tests (e.g., Peretz et al., 2003; Wallentin, Nielsen, Friis-Olivarius, Vuust, & Vuust, 2010). By contrast, ‘Singing Abilities’ and ‘Emotions’ had weaker but still significant correlations of a moderate magnitude. These weaker correlations with conceptually more distant subscales of the Gold-MSI are suggestive of the divergent validity of the TPT, at least when using self-report measures for comparison. Importantly, the TPT revealed considerably stronger correlation with the Gold-MSI compared with the tests from PSYCHOACOUSTICS toolbox and the Timbre subtest from PROMS. It implies that the newly developed instrument may be measuring an aspect of musical sophistication (i.e., an individual’s ability to perceive and reproduce timbre) more accurately than the previously published tests selected in this study.

The raw distances between the target and the performance values revealed that the accuracy to reproduce the timbre of tones is substantially reduced (see Table 3) when the playback of stimuli is limited compared with conditions where unlimited repetitions are possible. To further investigate this, future research on memory for timbre (e.g., Golubock & Janata, 2013; Halpern & Müllensiefen, 2008; see overview in Siedenburg & Müllensiefen, 2019) may benefit from implementing the TPT to investigate the decay of timbral memory by its independent dimensions, over multiple time periods.

The performance accuracy increased when participants chose to listen to more repetitions of the target stimulus and of the reproduction tones. One possible explanation for these correlations could be that the participants who were more uncertain took a multiple-look strategy. However, considering the strong correlations with the self-reported perceptual ability, the observed correlations between stimulus repetitions and task performance could also imply that the participants who were able to hear finer differences between the two tones, repeated the tones a greater number of times to make more fine-grained adjustments to the slider position. Though, interestingly, it was not the group of musically trained participants who showed greater efforts on the test, given that we observed no significant correlations between Musical Training subscale nor General Sophistication of the Gold-MSI with number of the TPT tone playbacks. Thus, this can be interpreted as an encouraging indication that the TPT may be an engaging and robust instrument to measure an individual’s perceptual abilities for timbre, regardless of their level of musical training.

Overall, the TPT has shown to be a promising tool for measuring individuals’ timbre perception ability. Additionally, its use of a production test paradigm and sliders to adjust timbral dimensions has the practical potential to combine short testing times with good measurement precision. This can lead to a greater test efficiency compared with traditional perceptual paradigms that can suffer from attentional lapses and fatigue due to the necessity to present a large number of trials to participants. Moreover, these individual responses only gain little information due to high guessing probabilities on 2AFC or same–different tasks. We propose that the TPT can be broadly applied in the field of perceptual psychology to address outstanding questions on the individual differences on timbre perception (Siedenburg & Müllensiefen, 2019). Furthermore, given that the test is provided as open source and its parameters can be easily manipulated, the testing paradigm does not only have to be restricted to assess timbre perception but applied to other aspects of auditory perception amenable to the employment of a production paradigm.

## Limitations and future works

There were several notable limitations to the current experiment. The use of a slider as a testing interface may have been limited in determining the precise perceptual thresholds of an individual. To validate this, a comparison between the results of a discrimination and the TPT reproduction task should be made in the future work. If measurements using the slider interface indeed lack in precision, an adaptive procedure could be implemented, whereby the auditory range of each dimension covered by the slider scale adaptively narrows or widens. Alternatively, borrowing the ideas from Turner et al. (1994), a hybrid model can be adopted in which the slider may be used to first determine the attentional focus and then quickly shifted to forced-choice procedure.

Two of the match items had to be excluded from the analysis as there was a substantial number of participants (>30%) who did not move the slider. These items were problematic because the target values were very near the default position of the slider (raw distances to the targets were less than 12 points on slider scale). The participants could have reasoned that the reproduction tone is already close enough to the stimulus tone even when the slider is left unmoved. This resulted in four trials (instead of five trials) for testing the matching variants of Envelope and Spectral Flux subtasks. Yet it is uncertain if the point of plateau has been reached at four trials on the Envelope and Spectral Flux subtasks because it was observed that a larger number of match trials leads to stronger correlations with the composite score of the Gold-MSI. Hence, a future version of the TPT may include a few more match trials with target values that are further away from the default position. A greater number of trials could also raise the internal consistency of the TPT even further.

The TPT uses synthetic sounds to avoid a common testing bias that favours musicians trained in Western art music. However, the exclusive use of synthetic sounds may have introduced a different kind of bias, possibly in favour of participants who mainly work with or listen to synthetic sounds. Hence in a future study, we aim to compare synthetic sounds with manipulable sounds from acoustical instruments within the TPT testing paradigm to examine the degree of dependency on the specific set of sounds employed. In a similar vein, the complex tone probe could be replaced—for instance, with a human voice–to examine the accuracy in perception of timbre of human vocal sounds against unfamiliar synthetic sounds. Some recent studies investigated timbre perception from an evolutionary angle inspired by the finding that the human voice is recognized much more quickly than instruments (e.g., Agus, Suied, Thorpe, & Pressnitzer, 2012; Suied, Agus, Thorpe, Mesgarani, & Pressnitzer, 2014). Thus, by adopting the TPT’s production paradigm, we can potentially compare the perceptual accuracy for vocal and synthetic sounds using a common framework of dimensions for timbre manipulation. In this respect, the TPT’s testing paradigm can serve as a starting point for addressing novel questions in interdisciplinary research.

Considering the weak loadings of memory variants on the single factor, the future version of the TPT will separate the match and memory variants, and it will be implemented online to enable the testing of larger and more diverse participant samples. When implemented online, we will look to present the items and task blocks in a random order given that order effect may have been present in the current study. Moreover, we plan to assess the divergent validity of the TPT with other auditory tests and nonauditory perceptual tests.

## Recommendation of use

Given the empirical results presented here, we recommend using the short version of the TPT for the inclusion into larger test batteries. The short version consists of four match trials for Envelope, four trials for Spectral Flux, and five trials for Spectral Centroid, taking about 8 minutes in duration. At the end of the test, the software outputs the acoustic parameter value of the target stimulus for each trial, the participant’s slider position, and number of playbacks of the target stimulus and reproduction tones. The short version of the TPT has an internal consistency of *α* = .69, ωt = .70 and a test–retest reliability of *ICC* (24) = .79, Pearson’s *r* = .79, *ρ* = .75. Psychometric indicators of validity can be found in Table 2. Nevertheless, the full version of the TPT including the memory trials is also openly available, taking about 15 minutes in duration, with internal consistency of *α* = .74, ωt = .80. The full version including the memory tasks may be useful for investigating questions regarding the encoding, storage and retrieval of timbre information from memory.

The openly available software (both versions can be downloaded at www.osf.io/9c8qz ) does not require any coding and runs as a standalone application on Windows (tested for Windows 10 and Windows 7; 32-bit and 64-bit) and Mac (tested for Version 10.13.6) operating systems. Conversion of the raw slider values reported in the output of the TPT application to bin scores can follow the bin boundaries of each item documented in Appendix C.

## Appendix A: Descriptive statistics of the full test battery

**Table 4:** Descriptive statistics of the TPT scores, thresholds of three tests from the PSYCHOACOUSTICS, PROMS(Timbre) score, and Gold-MSI

	Mean	*SD*	Median	Min.	Max.	*n*
***TPT*** *(scores out of 6, with 6 being best and 1 being worst performance)*						
Envelope (match)	3.668	1.090	3.750	1.000	6.000	95
Envelope (memory)	3.663	0.800	3.750	1.700	5.300	95
Spectral Flux (match)	3.628	0.940	3.500	1.500	5.750	95
Spectral Flux (memory)	3.611	0.619	3.650	1.500	5.100	95
Spectral Centroid (match)	3.632	0.973	3.600	1.200	5.400	95
Spectral Centroid (memory)	3.599	0.688	3.500	1.900	5.200	95
Match Total	3.642	0.792	3.692	1.692	5.385	95
Memory Total	3.624	0.443	3.650	2.533	4.567	95
Overall Score	3.629	0.501	3.640	4.567	4.814	95
***PSYCHOACOUSTICS toolbox*** *(thresholds)*						
Pitch Discrimination (Hz)	9.68	12.80	4.41	1.01	60.76	104
Duration Discrimination (ms)	39.44	26.69	32.13	10.54	203.77	103
Profile Analysis (dB)^1^	4.04	2.52	3.51	0.99	16.45	100
***PROMS*** *(scores out of 18)*						
Timbre subtest	11.32	2.17	11.00	6.00	17.00	104
***Gold-MSI***						
Active Engagement (G0)	42.47	9.76	44.00	17.00	61.00	104
Perceptual Abilities (G1)	48.60	8.99	48.00	23.00	63.00	104
Musical Training (G2)	26.96	12.34	27.00	7.00	49.00	104
Singing Abilities (G3)	34.21	4.85	34.00	23.00	42.00	104
Emotions (G4)	31.98	8.59	32.00	11.00	48.00	104
General Sophistication (G5)^2^	82.16	21.84	84.00	36.00	123.00	104

^1^Level of increase in sound intensity of the 3rd harmonic

^2^General Sophistication (G5) is the composite score of all subscales (G0–G4) of the Gold-MSI

## Appendix B: Additional correlations between tests within the battery

**Table 5:** Spearman’s correlation between PROMS Timbre subtest, Gold-MSI, and tests from PSYCHOACOUSTICS

	Gold-MSI						PSYCHOACOUSTICS		
	G0	G1	G2	G3	G4	G5	Pitch	Duration	Profile
PROMS (Timbre)	.30**	.37***	.38***	.33**	.35**	.42***	.31**	.28**	.18

G0 = Active Engagement; G1 = Perceptual Abilities; G2 = Musical Training; G3 = Singing Abilities; G4 = Emotions; G5 = General Sophistication. **p* < .05, ***p* < .01, ****p* < .001. Significant levels are adjusted according to Benjamini and Hochberg (1995)

**Table 6.** Spearman’s correlation between tests from PSYCHOACOUSTICS toolbox and Gold-MSI.

		Gold-MSI					
		G0	G1	G2	G3	G4	G5
PSYCHOACOUSTICS	Pitch	.44***	.36***	.56***	.33**	.19	.47***
	Duration	.31**	.18	.22*	.36**	.18	.24*
	Profile Analysis	.10	.18	.28**	.17	.19	.24*

G0 = Active Engagement; G1 = Perceptual Abilities; G2 = Musical Training; G3 = Singing Abilities; G4 = Emotions; G5 = General Sophistication. **p* < .05, ***p* < .01, ****p* < .001. Significant levels are adjusted according to Benjamini and Hochberg (1995)

## Appendix C: Bin boundaries and corresponding acoustic parameters

**Table 7:** Assigned bin scores by the lower and upper boundaries of six bins

Parameter manipulated in subtasks		Target value	Assigned bin scores by boundaries of bin categories ^1^					
			6	5	4	3	2	1
**Envelope** ^2^ (Slider range of 100 = 20–291 ms)	**Item 1**							
	Slider value	42	NA ^3^					
	Attack time (ms)	52.0						
	**Item 2**							
	Slider value	6	0–2	3–6	7–11	12–18	19–29	30–94
	Attack time (ms)	22.8	0.0–1.1	1.5–3.5	4.1–6.8	7.6–12	13–24	25–268
	**Item 3**							
	Slider value	60	0–1	2	3	4–6	7–11	12–60
	Attack time (ms)	92.8	0.0–3.6	5.0–5.4	7.4–8.1	9.8–17	16–34	26–198
	**Item 4**							
	Slider value	24	0–2	3–5	6–8	9–11	12–18	19–76
	Attack time (ms)	35.3	0.0–1.8	2.6–4.8	4.9–8.1	7.0–12	9.1–21	13–256
	**Item 5**							
	Slider value	77	0–1	2	3–4	5–9	10–19	20–77
	Attack time (ms)	150	0.0–4.4	8.0–9.0	12–18	20–44	37–109	65–145
**Spectral Flux** (Slider range of 100 = 0–0.25 of mean change in ratio of four harmonics)	**Item 1**							
	Slider value	60	0–5	6–10	11–12	13–20	21–28	29–60
	ß^4^	0.15	0.000–0.013	0.015–0.025	0.028–0.030	0.033–0.050	0.053–0.070	0.073–0.150
	**Item 2**							
	Slider value	24	NA					
	ß	0.06						
	**Item 3**							
	Slider value	77	0–5	6–11	12–15	16–23	24–33	34–77
	ß	0.19	0.000–0.010	0.013–0.030	0.028–0.040	0.038–0.060	0.058–0.080	0.083–0.190
	**Item 4**							
	Slider value	42	0–3	4–7	8	9–15	16–21	22–58
	ß	0.11	0.000–0.013	0.015–0.023	0.015–0.025	0.018–0.043	0.035–0.058	0.060–0.140
	**Item 5**							
	Slider value	94	0–16	17–24	25–33	34–44	45–48	49–94
	ß	0.24	0.000–0.040	0.043–0.060	0.063–0.083	0.085–0.110	0.113–0.120	0.123–0.240
**Spectral** **Centroid** (Slider range of 100 = 600–1000 Hz)	**Item 1**							
	Slider value	77	0–2	3–4	5–8	9–13	14–26	27–77
	Frequency (Hz)	837	0–14	18–25	30–52	52–87	78–109	93–237
	**Item 2**							
	Slider value	42	0–7	8	9–12	13–19	20–31	32–58
	Frequency (Hz)	671	0–25	25–29	27–46	37–78	52–142	67–329
	**Item 3**							
	Slider value	94	0–2	3–5	6–7	8–11	12–19	20–94
	Frequency (Hz)	953	0–16	22–39	43–50	57–77	84–128	134–353
	**Item 4**							
	Slider value	60	0–4	5–7	8–10	11–13	14–22	23–60
	Frequency (Hz)	744	0–20	23–36	36–52	48–69	59–125	89–256
	**Item 5**							
	Slider value	6	0–14	15–25	26–38	39–44	45–51	52–94
	Frequency (Hz)	601	0–15	17–37	40–76	80–99	103–129	134–399

^1^Boundaries of bin categories are represented as absolute distance from the target of slider value (with a theoretical range 0–100) and corresponding acoustic parameter

^2^Although both log attack and decay were manipulated in the Envelope subtask, only the attack threshold is reported because this was the parameter of interest

^3^NA, item excluded due to more than 30% of participants not moving the slider

^4^ß = arithmetic mean deviation in ratio of four harmonics from their original whole number integers

## Footnotes

1 Online hearing test: www.hear-it.org
